# Compensation of Dynamic Fixation Systems in the Quality of Reduction of Distal Tibiofibular Joint in Acute Syndesmotic Complex Injuries: A CT-Based Analysis

**DOI:** 10.1177/10711007221115193

**Published:** 2022-08-09

**Authors:** Fabian T. Spindler, Federico P. Gaube, Wolfgang Böcker, Hans Polzer, Sebastian F. Baumbach

**Affiliations:** 1 Department of Orthopaedics and Trauma Surgery, Musculoskeletal University Center Munich (MUM), University Hospital, LMU Munich, Munich, Germany

**Keywords:** suture button system, syndesmosis, stabilization

## Abstract

**Background:**

There is an ongoing discussion on how to best stabilize syndesmotic injuries. Previous studies have indicated a better quality of reduction of the distal tibiofibular joint (DTFJ) for the suture button systems compared to syndesmotic screw fixation. Still, the reason for this superiority remains unclear. The aims of this retrospective study were to (1) analyze the deviation of the tibial and fibular drilling tunnels of the suture button system and (2) to compare these to the quality of reduction of the DTFJ assessed on bilateral postoperative CT images.

**Methods:**

Included were all adult patients who underwent syndesmotic stabilization for an acute injury using a suture button system, with postoperative, bilateral CT imaging over a 10-year period. A total of 147 patients were eligible. Based on individually reconstructed axial CT slices, the postoperative quality of reduction of the DTFJs was rated on bilateral CT images. Furthermore, the rotation and translation of the suture button drilling tunnels were analyzed. Based on these measurements, the intraoperative reduction of the DTFJ was recalculated and again rated. Using these values, the correction potential of suture button systems on the reduction of the DTFJ was analyzed.

**Results:**

(1) The drilling tunnel deviated considerably for both rotation |2.3±2.1 degrees| (range: |0.0-13.1 degrees|) and translation |0.9±0.8 mm| (range: |0-4.3 mm|). Based on the deviation of the drilling tunnels in fibula and tibia, the calculated intraoperative reduction of the DTFJ was classified as malreduced in 35.4%. (2) The DTFJ was postoperatively identified as malreduced in 17% of patients. Overall, the suture button system tended to compensate toward a more anatomical reduction both in the axial and sagittal plane.

**Conclusion:**

A suture button system postoperatively deviates and apparently has the capacity to compensate for intraoperative malreduction. Analysis of the drilling tunnels revealed that the use of a rigid fixation system would have doubled the postoperative malreduction rate.

**Level of Evidence:** Level III, retrospective radiological study.

## Introduction

Syndesmotic injuries occur in up to 17% of all ankle sprains,^11,15^ increasing to 30% in high-impact sports.^25^ Furthermore, about 20% of all ankle fractures necessitate surgical stabilization of the syndesmotic complex.^9,22^ Neglected or malreduced syndesmotic injuries result in considerable impairment, including instability, pain, and osteoarthritis.^1,13,32^ Sagi et al^34^ compared the patient-rated outcome between anatomically reduced and malreduced distal tibiofibular joints (DTFJs) 2 years after surgery and found a significant differences for the Short Form Musculoskeletal Assessment functional score (12.0 vs 27.0 points, *P* < .05) and for the Olerud-Molander Ankle Score (46.3 vs 72.2 points; *P* < .05). Andersen et al^2^ used mixed effects linear regression models to correlate the degree of DTFJ reduction assessed on bilateral CT images (postoperatively, at 1- and 2-year follow-up) and the patient-rated outcome (Olerud-Molander Ankle Score, American Orthopaedic Foot & Ankle Society ankle/hindfoot scale). For the anterior tibiofibular distance, a 1-mm widening of the injured compared to the uninjured ankle resulted in a mean reduction of the Olerud-Molander Ankle Score / American Orthopaedic Foot & Ankle Society score of 2.6/2.2 points. Despite the importance of an anatomically reduced DTFJ, studies suggest syndesmotic malreduction rates of up to 39%.^16,21,35,37,40^

Consequently, there is an ongoing discussion on the value of suture button systems compared to syndesmotic screws. Biomechanical studies were able to reveal that syndesmotic screws provide greater primary stability, but rigidly fix the fibula to the distal tibia. Suture button systems on the other hand provide a dynamic and possibly more physiological fixation.^5,31,33^ Moreover, these studies have also indicated that a suture button system could compensate for intraoperative malreduced DTFJs.^29,38^

Numerous randomized controlled trials have compared the clinical outcome of suture button systems to syndesmotic screw fixation.^3,7,17,19,21,29,30,35^ For both the patient-reported outcomes and the quality of reduction, most studies have pointed to a possible superiority of the suture button system.^3,17,19,29,35^ One reason could be their above-outlined natural compensation potential for intraoperatively malreduced DTFJs,^38^ also referred to as self-centering theory.^29^ From our own experience, the drilling tunnels of the suture button in the tibia and fibula often deviates on postoperative CT scans, whereas the DTFJ is still anatomically reduced. Until now, no study has investigated this possible compensatory mechanism of suture button systems in a large clinical cohort.

Therefore, the goal of this retrospective study was first to analyze the deviation of the tibial and fibular drilling tunnels and second to compare these to the quality of reduction of the DTFJ assessed on bilateral postoperative CT images.

## Materials and Methods

This retrospective radiographic study was approved by the local ethics committee (#21-1136).

### Patient Selection

Patients who underwent syndesmotic stabilization at a single academic, level 1 trauma center between January 2010 and October 2020 were identified by the specific OPS-Code 5-869. Eligible were all adult patients (>18 years of age) who underwent surgical stabilization of the syndesmosis with a suture button system (TightRope; Arthrex, Naples, FL) for an acute, unilateral ankle injury with postoperative, bilateral CT scans. The type of injury, that is, an ankle fracture or isolated syndesmotic instability, were of no matter. Excluded were any patients with known injuries to the contralateral ankle. At the authors’ institution, syndesmotic stability is intraoperatively tested either by the external rotation stress test, or the hook test. The reduction of the DTFJ is performed by reduction forceps, placed in neutral axis on the medial and lateral malleolus. The decision on the implant, that is, syndesmotic screw or a suture button system, was up to the surgeon. Independent of the device used, it was placed just proximal to the articulation between the fibula and tibia and whenever possible through one of the plate holes. Following surgery, patients were instructed to use crutches for 6 weeks of partial weightbearing (20 kg). Postoperative, bilateral CT imaging must have been conducted within the first week after surgery.

### Imaging Analysis

The bilateral CT scans were conducted on different scanners, with a varying slice thickness between 0.65 and 2 mm. All imaging analysis were conducted on postoperative, bilateral CT images using the VisageCS-Client, version 7.1.16 (Visage Imaging Inc, San Diego, CA). All measurements were conducted by one investigator (F.T.S.) per a standardized protocol, photographically documented, and reviewed by the senior author (S.F.B.). In case of disagreement, the measurements were reconducted.

**Figure 1.**
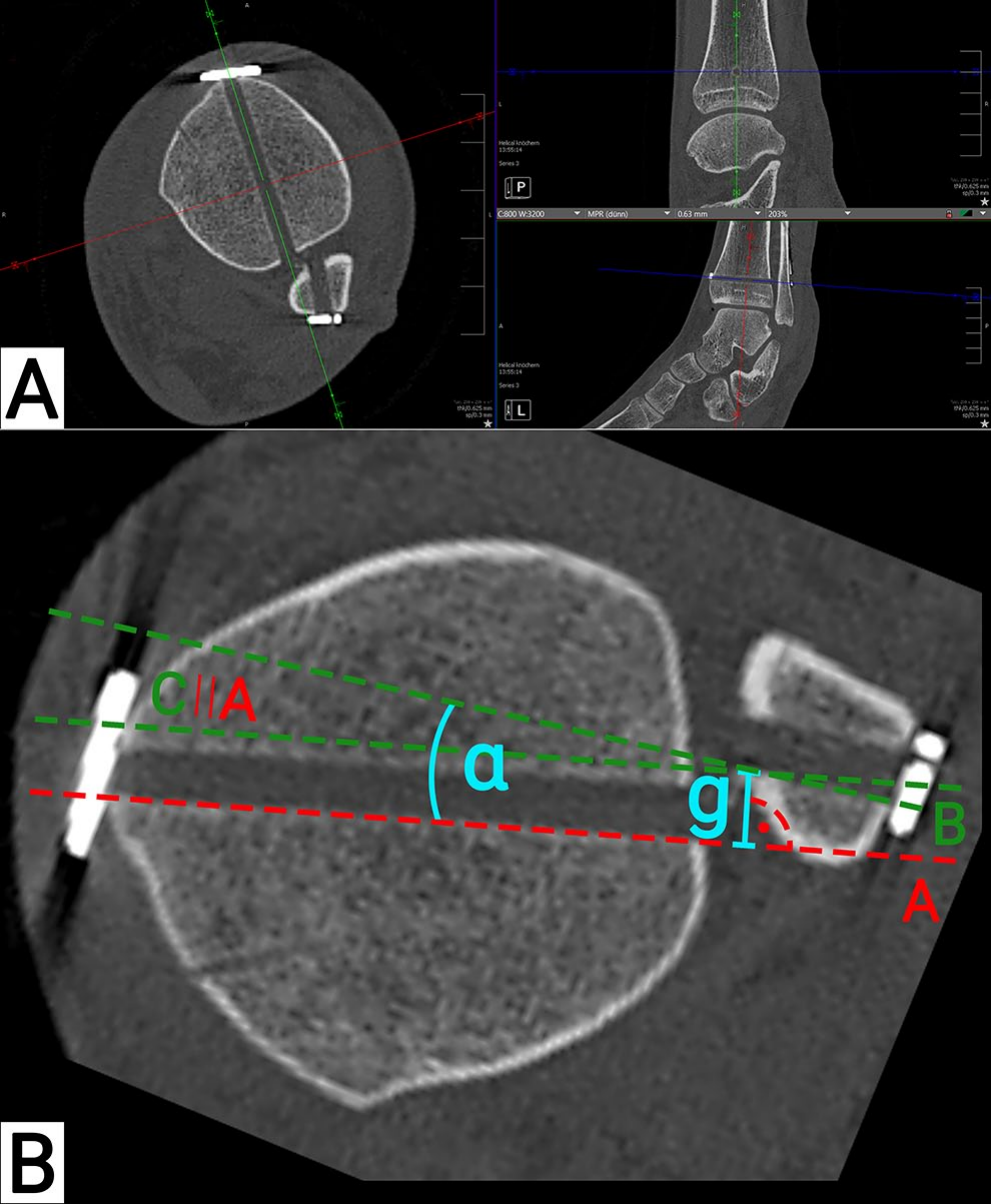
Suture button drilling tunnel measurements. (A) Computed tomographic reconstructions of the drilling tunnel; (B) measurement of the drilling tunnel. A: straight line on the posterior margin of the tibial drilling tunnel; B: straight line on the posterior margin of the fibula drilling tunnel; C: translation of A to the posteromedial edge of the fibular drilling tunnel; α: angle between the tibial and fibular drilling tunnel; g: translation between the tibial and fibular drilling tunnel; ∣∣: translation.

Each CT data set was reconstructed separately. For the analysis of the suture button drilling tunnel, axial CT slices were generated showing the entire drilling tunnel, that is, within the tibia and fibula, at its maximum diameter (Figure 1A). Assessed were the rotation (α; Figure 1B) and translation (g; Figure 1B) of the drilling tunnels between the tibia and fibula. Both measurements were conducted on the posterior border of the tibial and fibular drilling tunnel. For rotation, negative values indicated an internal, positive values and external rotation of the fibula. For translation, an anterior translation of the fibula was indicated by positive, and a posterior translation by negative values.

**Figure 2.**
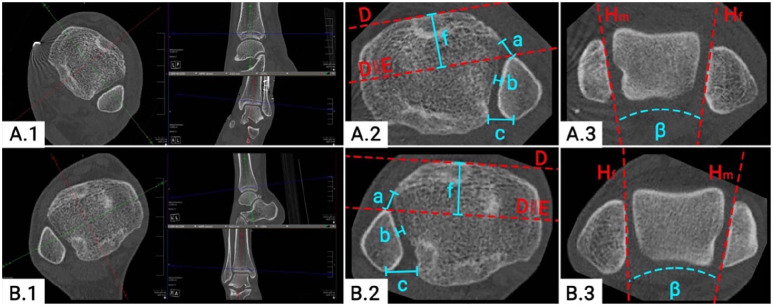
Quality of distal tibiofibular joint (DTFJ) reduction on bilateral computed tomographic images. (A.1) DTFJ reconstruction of the reduced ankle; (B.1) DTFJ reconstruction of the contralateral side; (A.2) measurement of the DTFJ reduction of the reduced ankle; (B.2) measurement of the DTFJ reduction of the contralateral ankle; (A.3) Nault talar dome angle (NTDA) of the reduced ankle; (B.3) NTDA of the contralateral side. D: tangent to the anterior aspect of the Tibia; E: translation of D to the most anterior part of the fibula; H: tangent to the (m) medial malleolus / (f) fibula; a: anterior tibiofibular distance; b: central tibiofibular distance; c: posterior tibiofibular distance; f: sagittal DTFJ translation of the reduced ankle; β: NTDA; ∣∣: translation.

The quality of reduction of the DTFJ was again assessed on separately reconstructed axial slices, independent for both ankle joints (Figure 2A.1, B.1). Measurements were conducted on the first distal tibial slice with no subchondral bone visible. Assessed were the anterior, central, and posterior distance between the tibia and fibula, as recommended by Kubik et al^18^ (a, b, and c; Figure 2A, B.2). Fibular rotation was measured by the Nault talar dome angle (NTDA) (β; Figure 2A, B.3). Sagittal translation of the fibula was assessed using a modification of the technique described by Prior et al^28^ (h; Figure 2A, B.2). All measurements were conducted bilaterally.

### Data Assessed

Demographic details, including age, sex, height, weight, BMI, and ASA classification, were assessed. The injuries were classified as fractures or isolated syndesmotic injuries.

For all bilateral radiographic measurements assessed, the difference (delta values) between the injured and uninjured sides were calculated. Each DTFJ was classified as anatomically reduced or malreduced based on the recommendations by Kubik et al.^18^ Malreduction was defined as an anterior-posterior tibiofibular distance >2 mm, a central tibiofibular distance >1.5 mm, a translation >2 mm, or a NTDA >10 degrees. If malreduced, the predominant direction of malreduction was assessed, that is, rotational, translational, combined, overcompressed, or ungroupable.

**Figure 3.**
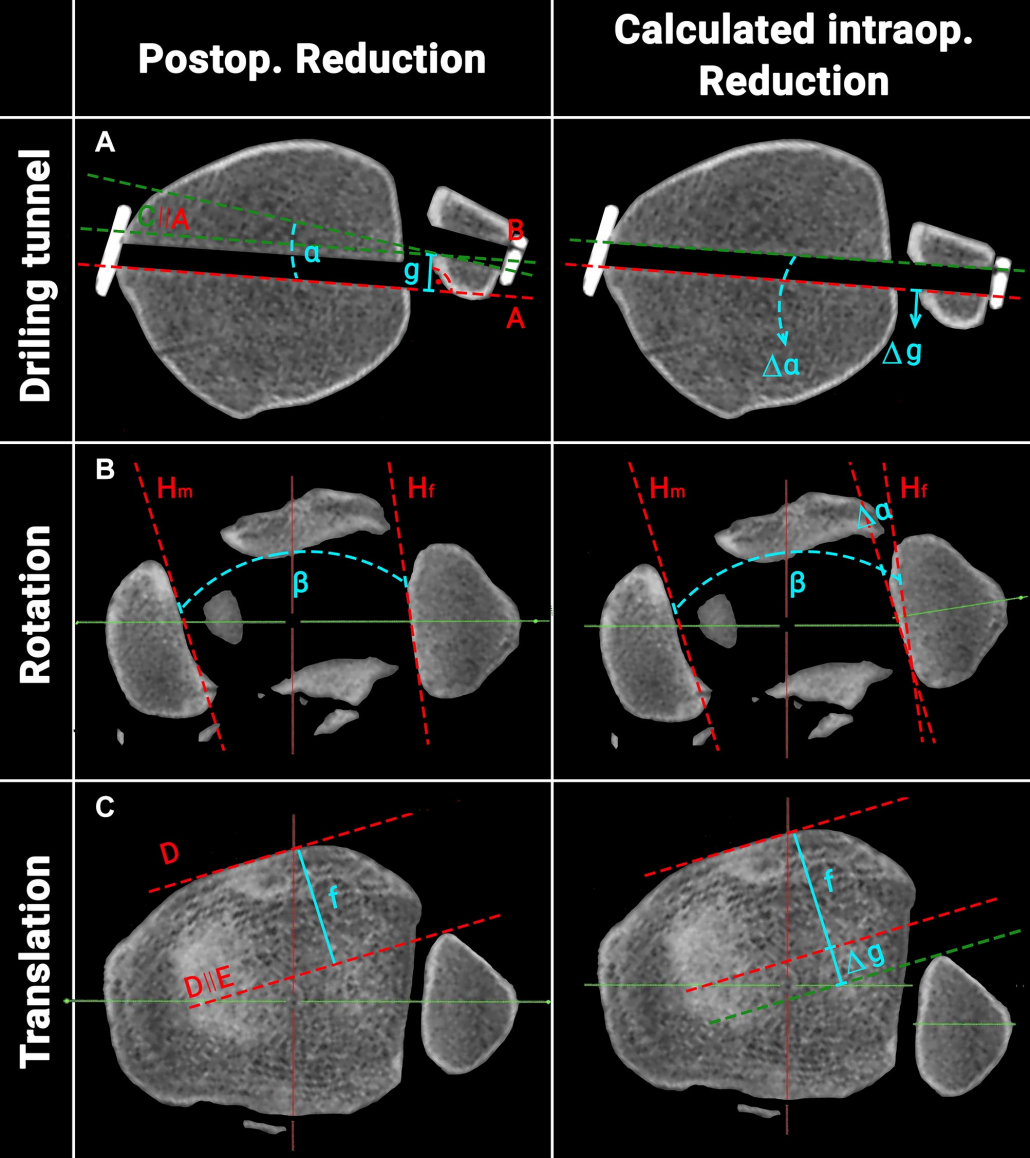
Schematic illustration of the process to calculate the intraoperative reduction. A: Straight line on the posterior margin of the tibial drilling tunnel; B: straight line on the posterior margin of the fibula drilling tunnel; C: translation of A to the posteromedial edge of the fibular drilling tunnel; H: tangent to the (m) medial malleolus / (f) fibula; α: angle between the tibial and fibular drilling tunnel; g: translation between the tibial and fibular drilling tunnel; ∣∣: translation. (Postop.: postoperative; Intraop.: intraoperative.)

Next, the intraoperative reduction at the time of drilling for the suture button system was calculated. The process is outlined in Figure 3 and based on the assumption that the suture button drilling tunnels within the tibia and fibula must have been aligned (drilling tunnel; Figure 3). In summary, the previously assessed rotation (α; Figure 3) and translation (g; Figure 3) of the drilling tunnels between the tibia and fibula were subtracted/added from/to the ipsilateral postoperative NTDA (β; Figure 3) and sagittal translation measurements (f; Figure 3). Then, these corrected values were again compared to the uninjured, contralateral side and the quality of DTFJ reduction reassessed, per the criteria outlined above.

### Statistics

Statistical analysis was performed using IBM Statistical Package for the Social Sciences, version 28. Because of the retrospective nature of the study, no a priori power analysis was performed. Next to descriptive statistics, independent samples t tests and χ^2^ tests were used. Values are given as mean ± standard deviation. *P* values lower than .05 were considered statistically significant.

## Results

### Patient Selection

**Figure 4.**
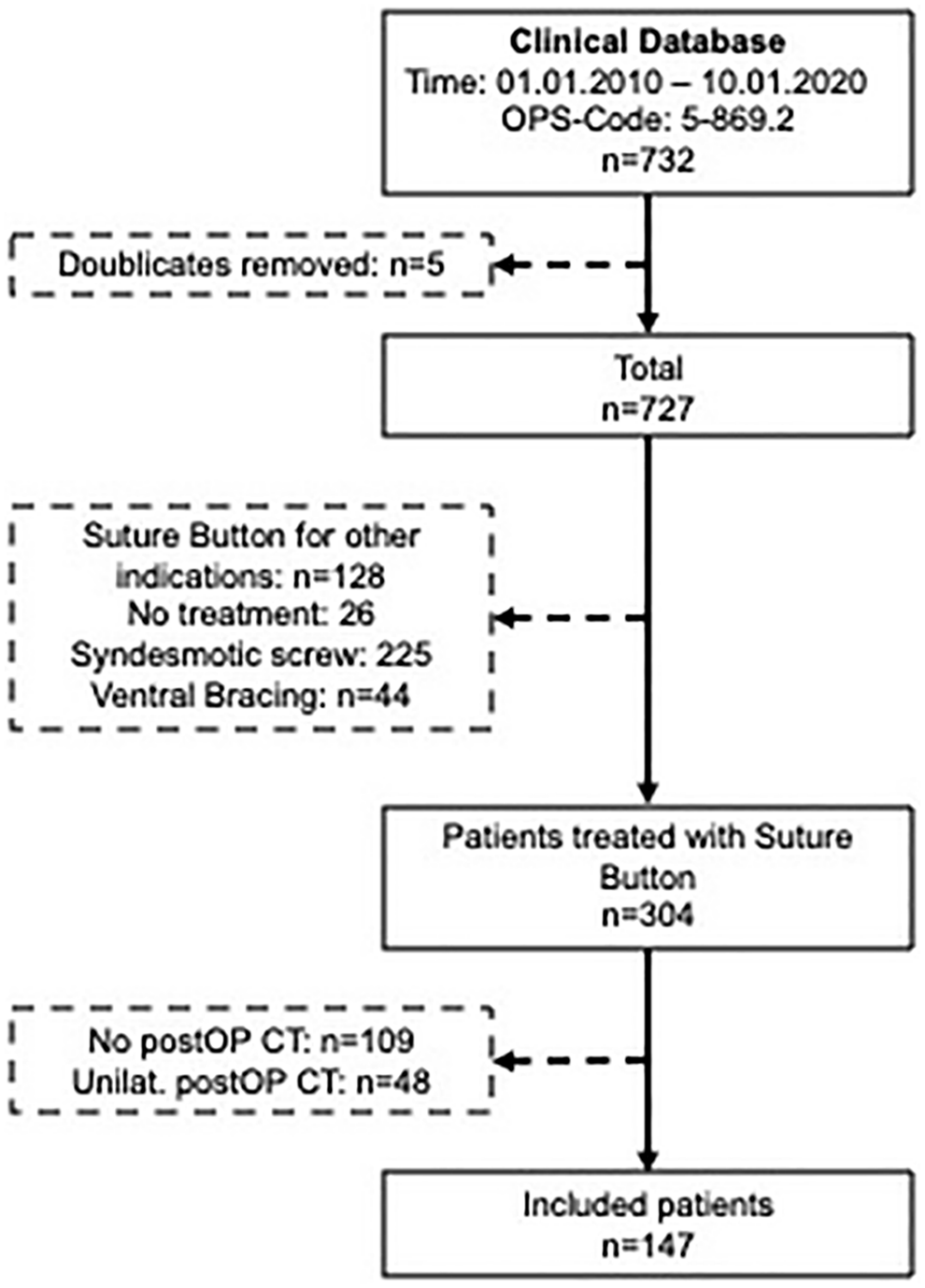
Patient selection flow chart. CT, computed tomography; n, number of patients; postOP, postoperatively; Unilat., unilateral.

The patient selection flowchart is presented in Figure 4. Out of 304 patients treated with a suture button system for syndesmotic instability over the last decade, 147 were eligible. The first suture button system was implanted in January 2014. The patients’ mean age was 39.3±14.8. Their mean BMI was 26.3±4.8, 34% were female and the left side was affected in 50%. The primary injury was an ankle fracture in 63.3%. The remaining patients suffered an isolated syndesmotic instability.

### Suture Button Drilling Tunnel

**Figure 5.**
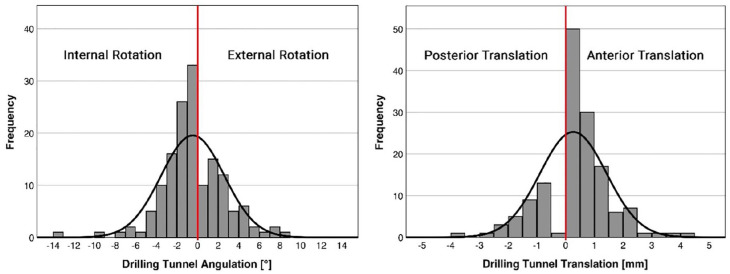
Rotation and translation of the suture button drilling tunnel of all patients.

The drilling tunnel, in its maximum diameter, could be reconstructed in all cases. The rotation and translation of the drilling tunnel is presented in Figure 5. Overall, the mean absolute values of the drilling tunnels for rotation were |2.3±2.1 degrees| (range: |0.0-13.1 degrees|). Two patients (1.4%) had 0 degrees of rotation, 64.6% of the drilling tunnels had an external rotation at an average of 2.2±2.1 degrees (range: 0.1-13.1 degrees), and 34.0% were rotated internally at an average of 2.7±2.1 degrees (range: 0.1-8.6 degrees). The mean absolute values for translation in the sagittal plane were |0.9±0.8 mm| (range: |0-4.3 mm|). For translation, 41 patients (27.9%) had no translation (0 mm), in 49.7% the drilling tunnel revealed an anterior translation with a mean of 1.1±0.8 mm (range: 0.2-4.3 mm) and in 22.4% a posterior translation of 1.3±0.7 mm (range: 0.5- 3.6 mm).

### Quality of DTFJ Reduction

A total of 122 patients (83%) had an anatomically reduced DTFJ, based on the postoperative, bilateral, axial CT measurement. The DTFJ was formally malreduced in 25 patients (17%). There was no significant difference per the patients’ ages (39.2±14.6 years vs 39.8±16.4 years; *P* = .838) or BMI (26.3±4.87 vs 25.9±4.4; *P* = .757) between anatomically and malreduced patients. Malreduction occurred significantly more often in fracture cases, compared with isolated syndesmotic injuries (24.7% vs 3.7%; *P* = .005). Malreduction was predominantly rotational in 6 patients (24%; internal rotation: 17%; external rotation: 83%) and translational in 9 patients (36%; anterior translation: 44%; posterior translation: 56%). Three patients (12%) had a combined malrotation and translation, 3 patients (12%) an overcompression, and 4 patients (16%) did not fit in any group. Five patients (3.4%) underwent revision surgery; the remaining were not revised.

### Comparison of Intraoperative and Postoperative DTFJ Reduction

Based on the calculated intraoperative DTFJ reduction, 95 DTFJs (64.6%) were anatomically reduced and 35.4% malreduced (Table 1). Fixing the fibula by a rigid device, that is, in the position when tibial and fibular drilling tunnels were aligned, would therefore have doubled the malreduction rate (17% vs 35.4%).

**Table 1.** DTFJ Reduction per TightRope and Adjusted for Drilling Tunnel.

	DTFJ Anatomical, n (%)	DTFJ Malreduced, n (%)	Total
Postoperative DTJF reduction	122 (83.0)	25 (17.0)	147
Calculated intraoperative DTFJ reduction	95 (64.6) (+n=1)	51 (35.4) (+n=28)	147

Abbreviation: DTFJ, distal tibiofibular joint.

**Figure 6.**
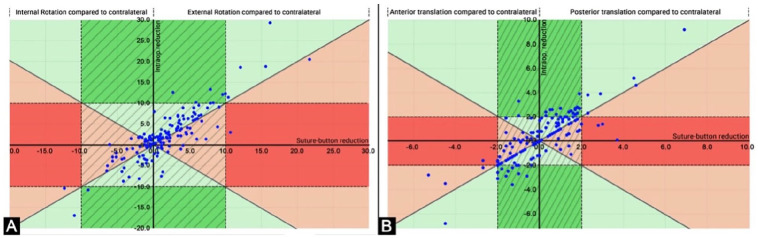
Scatterplot for the distal tibiofibular joint (DTFJ) reduction intraoperatively vs postoperatively separately for (A) rotation and (B) translation. A: rotation; B: translation; hatched area: anatomically reduced with the suture button system; green areas: postoperative DTFJ reduction better than calculated intraoperative reduction with forceps; red areas: postoperative DTFJ reduction worse than calculated intraoperative reduction with forceps; dark green areas: DTFJs that were intraoperatively malreduced but postoperatively anatomically reduced; dark red areas: DTFJs that were intraoperatively anatomically reduced and postoperatively malreduced.

The differences between intraoperative and postoperative DTFJ reduction is illustrated as a scatter plot in Figure 6, separately for rotation (Figure 6A) and translation (Figure 6B). The postoperative DTFJ rotational reduction improved in 95 patients (64.6%; Figure 6A, green areas) and worsened in 48 patients (32.7%; Figure 6A, red areas) compared to the intraoperative reduction. Four patients (2.7%) were rotationally malreduced intraoperatively, but revealed an anatomical rotational reduction postoperatively (Figure 6A, dark green area). One patient (0.7%) converted from intraoperatively anatomically reduced to postoperatively malreduced (Figure 5A, dark red area).

The postoperative DTFJ translational reduction improved in 71 patients (48.3%; Figure 6B: green areas) and worsened in 23 patients (15.6%; Figure 6B: red areas) compared to the intraoperative reduction. In 42 patients (28.6%), there was no difference. Twenty-three patients (15.6%) were translationally malreduced intraoperatively but revealed an anatomical rotational reduction postoperatively (Figure 6B, dark green area). Four patients (2.7%) converted from intraoperatively anatomically reduced to postoperatively malreduced per the translation (Figure 6B, dark red area).

## Discussion

This is the first study to analyze the effect of a suture button system on the quality of reduction of the DTFJ in a large clinical cohort based on postoperative, bilateral CT images. The drilling tunnel postoperatively revealed a considerable rotational and translational deviation in almost all cases. When comparing the calculated intraoperative to the postoperative quality of reduction of the DTFJ, the malreduction rate decreased considerably from 35.4% intraoperatively to 17% postoperatively. Overall, the suture button system tended to compensate toward a more anatomical reduction for both rotation and translation.

Previous studies were able to show that DTFJ malreduction is an independent risk factor for an impaired patient-rated outcome.^2,34^ Meta-analysis indicated a lower rate of DTFJ malreduction for the suture button system, compared with syndesmotic screws.^23,36^ Today, the authors are aware of 8 RCTs comparing suture button and syndesmotic screw fixation, with 1 study^21^ presenting even a 5-year follow-up.^17^ When evaluating the clinical outcome of these 8 randomized controlled trials, 3 studies found no differences^17,21,30^ and 5 studies reported superior outcomes for the suture button system for at least 1 clinical outcome parameter.^3,7,29,35^ Whether these superior results are because of a higher quality of reduction of the DTFJ remains unknown. Still, 4 studies reported on the postoperative malreduction rate based on bilateral CT imaging.^3,19,30,35^ Three studies reported lower malreduction rates for the suture button group,^3,29,35^ and 1 study found no differences.^30^ The herein presented study highlights the previously proposed correction potential of the suture button system for both rotation and translation.

The current study did not only assess the quality of reduction of the DTFJ on bilateral CT images but also analyzed the axial and sagittal deviation of the suture button drilling tunnels. As all drilling tunnels could be reconstructed on axial slices in their maximum diameters, there apparently was no relevant coronal translation between the drilling tunnels in the tibia and fibula. Two biomechanical studies compared the postoperative DTFJ alignment following syndesmotic screw or suture button stabilization in clamp-induced DTFJ malreduction models.^14,38^ Honeycutt and Riehl^14^ assessed the correction potential of a suture button construct (TightRope; Arthrex) for sagittal malreduction, that is, anterior or posterior translation, Westermann et al^38^ assessed sagittal and coronal (medial to lateral) malreduction. For both studies, the suture button system resulted in an anatomical reduction for anteriorly and posteriorly malreduced DTFJs. The authors are not aware of any study assessing rotational malreduction. This natural correction potential, not only for translation but also for rotation, also became evident in the current in vivo study.

Honeycutt and Riehl^14^ hypothesized that this compensatory mechanism is due to a size mismatch between the diameter of the sutures (6 FiberWires no. 5, 0.7 to 0.8 mm per FiberWire) and the size of the drilling tunnel (3.7 mm).^14^ This size mismatch would theoretically result in a maximum translation $\left(3.7\mathrm{mm}-\left(\sqrt[{2}]{{\left(0.75/2\right)}^{2}*\pi *6)/\pi }*2\right)\right)$ between 1.7 and 2.0 mm. However, in the current study, 13% and 10% of the measurements revealed a translation greater than 1.7 and 2.0 mm, respectively. Consequently, the compensatory translation can not only be explained by a size mismatch but also an additional correction in the coronal plane. Possible factors affecting the direction and degree of compensation are the depth of the concavity of the tibial incisura and the intraoperatively applied compression. Finally, the initial degree of tightening of the suture button system might also have an influence on its compensational capacity.

Despite the herein described correction potential of a suture button system, 25 DTFJs (17%) were formally malreduced, 5 of which were revised. The decision for revision was made by the treating surgeon. Still, it has been shown that based on the herein applied rigorous cutoff values, up to 35% of healthy ankles are formally considered malreduced for at least 1 parameter.^18^ Moreover, more recent studies on 4-dimensional CT imaging have demonstrated a subtle, ankle joint position–dependent deviation of the DTFJ.^39^ Furthermore, in 7 of the remaining 20 cases, there was an additional malreduction of the fracture which, to the treating surgeon’s opinion, was no indication for revision. Of the remaining 13 cases, 1.5±0.5 of 5 measurements conducted were above the cutoff values, whereas the other values were within the normal range.

Another reason for DTFJ malreduction could be the applied intraoperative reduction technique. Published techniques included manual (thumb) reduction,^8,20^ the glide path technique,^12^ or the clamp technique.^4,24^ Up to now, there is no agreement on the most appropriate reduction technique.^26^ The use of a clamp has the advantage that it fixes the DTFJ in its reduced position, and the surgeon can easily perform the stabilization. However, reduction forceps, if not used correctly, bear especially the risk to malrotate the fibula in the tibial incisura.^24,27^ However, the risk of malrotation can be minimized if the clamp is applied in a neutral anatomical axis.^8,27^ The current analysis revealed that the DTFJs were intraoperatively predominantly malreduced in internal rotation and posterior translation. As a result of the considerably high intraoperative malreduction rate, the authors have started to also directly visualize the anterior aspect of the DTFJ.^8^

Several limitations of this study have to be discussed. First, DTFJ measurements were not conducted 1 cm proximal to the joint line, as done in most previous studies,^18^ but on the first axial CT slice with no subchondral bone visible. This was done to account for intersubject variance. It can be assumed that 1 cm proximal to the joint line refers to a different anatomical region in patients with 150 cm compared to 200 cm of body height. Second, only 1 investigator (F.T.S.) conducted the radiographic measurements. Still, each measurement was photographically documented and reviewed by the senior author (S.F.B.). Often, measurements are conducted by 2 investigators and averages are calculated. Although the measurement locations are clearly defined in literature, intersubject anatomical variability does not always allow to identify identical landmarks, which would considerably alter the results. Instead, the authors followed the 4-eyes principle by reviewing each measurement based on the photographic documentation. Second, the search time frame chosen was January 2010 to October 2020. During the patient selection process, it became apparent that not until 2014 the suture button system had become the predominant stabilization device in our institution. The postoperative imaging has also evolved from radiographs or unilateral CT imaging to bilateral CT imaging. Although novel techniques facilitating conventional radiographs^6^ or intraoperative CT imaging have been postulated,^10^ bilateral CT imaging has progressively been applied at our institution since 2015. Although the initial search time frame was therefore too large, the authors’ intention was not to miss any patient. Finally, the calculated intraoperative DTFJ reduction is only an approximation of the actual intraoperative DTFJ reduction as it was solely based on rotation and translation. It did not allow to conclude on the anterior, central, or posterior tibiofibular distance measurements. Still, the current study was able to prove that the correction of a suture button system is multidimensional and does not result only from a mismatch between the sutures diameter and the drilling tunnel size.

Although previous biomechanical and clinical studies indicated a natural correction potential of the DTFJ for suture button systems, this is the first study to show this correction potential in a large clinical sample. The direction of correction was largely toward an anatomical DTFJ reduction and predominantly translational and rotational. The analysis of the drilling channels revealed that a rigid fixation method would have increased the malreduction rate by more than 100%.

## Supplemental Material

sj-pdf-1-fai-10.1177_10711007221115193 – Supplemental material for Compensation of Dynamic Fixation Systems in the Quality of Reduction of Distal Tibiofibular Joint in Acute Syndesmotic Complex Injuries: A CT-Based Analysis Click here for additional data file. Supplemental material, sj-pdf-1-fai-10.1177_10711007221115193 for Compensation of Dynamic Fixation Systems in the Quality of Reduction of Distal Tibiofibular Joint in Acute Syndesmotic Complex Injuries: A CT-Based Analysis by Fabian T. Spindler, Federico P. Gaube, Wolfgang Böcker, Hans Polzer and Sebastian F. Baumbach in Foot & Ankle International
